# The Pore-Forming Protein Cry5B Elicits the Pathogenicity of *Bacillus* sp. against *Caenorhabditis elegans*

**DOI:** 10.1371/journal.pone.0029122

**Published:** 2011-12-22

**Authors:** Melanie F. Kho, Audrey Bellier, Venkatasamy Balasubramani, Yan Hu, Wayne Hsu, Christina Nielsen-LeRoux, Shauna M. McGillivray, Victor Nizet, Raffi V. Aroian

**Affiliations:** 1 Division of Biological Sciences, Section of Cell and Developmental Biology, University of California San Diego, La Jolla, California, United States of America; 2 Department of Pediatrics, University of California San Diego, La Jolla, California, United States of America; 3 Skaggs School of Pharmacy and Pharmaceutical Sciences, University of California San Diego, La Jolla, California, United States of America; 4 Institut National de la Recherche Agronomique (INRA), UMR 1319 MICALIS, Guyancourt, France; 5 Department of Biology, Texas Christian University, Fort Worth, Texas, United States of America; Duke University Medical Center, United States of America

## Abstract

The soil bacterium *Bacillus thuringiensis* is a pathogen of insects and nematodes and is very closely related to, if not the same species as, *Bacillus cereus* and *Bacillus anthracis*. The defining characteristic of *B. thuringiensis* that sets it apart from *B. cereus* and *B. anthracis* is the production of crystal (Cry) proteins, which are pore-forming toxins or pore-forming proteins (PFPs). Although it is known that PFPs are important virulence factors since their elimination results in reduced virulence of many pathogenic bacteria, the functions by which PFPs promote virulence are incompletely understood. Here we study the effect of Cry proteins in *B. thuringiensis* pathogenesis of the nematode *Caenorhabditis elegans*. We find that whereas *B. thuringiensis* on its own is not able to infect *C. elegans*, the addition of the PFP Cry protein, Cry5B, results in a robust lethal infection that consumes the nematode host in 1–2 days, leading to a “Bob” or bag-of-bacteria phenotype. Unlike other infections of *C. elegans* characterized to date, the infection by *B. thuringiensis* shows dose-dependency based on bacterial inoculum size and based on PFP concentration. Although the infection process takes 1–2 days, the PFP-instigated infection process is irreversibly established within 15 minutes of initial exposure. Remarkably, treatment of *C. elegans* with Cry5B PFP is able to instigate many other *Bacillus* species, including *B. anthracis* and even “non-pathogenic” *Bacillus subtilis*, to become lethal and infectious agents to *C. elegans*. Co-culturing of Cry5B-expressing *B. thuringiensis* with *B. anthracis* can result in lethal infection of *C. elegans* by *B. anthracis*. Our data demonstrate that one potential property of PFPs is to sensitize the host to bacterial infection and further that *C. elegans* and probably other roundworms can be common hosts for *B. cereus*-group bacteria, findings with important ecological and research implications.

## Introduction

The *Bacillus cereus* group of bacteria comprises six species, including three highly related species, *B. cereus sensu stricto*, *B. anthracis* and *B. thuringiensis*, which are sometimes considered a single species [1], [2], [3]. Although closely related, these bacteria are associated with very different diseases. *B. anthracis* is the causative agent of anthrax [4], *B. cereus* can cause food poisoning and various opportunistic and nosocomial infections [5], and *B. thuringiensis* is an invertebrate-specific pathogen [6]. The relationship between these three closely related species of *Bacillus* in the wild, *e.g.* how they replicate in the soil environment and what environmental niches they occupy relative to one another, has been the subject of much speculation [7], [8].

The defining characteristic of *B. thuringiensis* is the production of Crystal (Cry) proteins, a large family of related proteins that kill insects and nematodes [8], [9], [10]. Each *B. thuringiensis* strain found in the wild can produce one or multiple (typically 2–4) Cry proteins. These Cry proteins are pore-forming proteins (PFPs), and thus are members of the single largest class of bacterial virulence factors [11]. Indeed, roughly 25–30% of all protein toxins made by human bacterial pathogens are PFPs, many with proven roles in disease pathogenesis. Purified Cry proteins alone, even in the absence of *B. thuringiensis*, produce dose-dependent lethality in invertebrates, and as such can be expressed in transgenic crops to control insect pests.

Here for the first time we characterize in detail the effect of Cry protein PFP administration on the outcome of *B. thuringiensis* infection in the nematode *Caenorhabditis elegans*, and uncover an unexpected interaction of Cry proteins with other *Bacillus* species bacteria during *C. elegans* infection.

## Results

### Infectivity of *C. elegans* by *Bacillus* species in presence of Cry5B pore-forming protein

**Figure 1:**
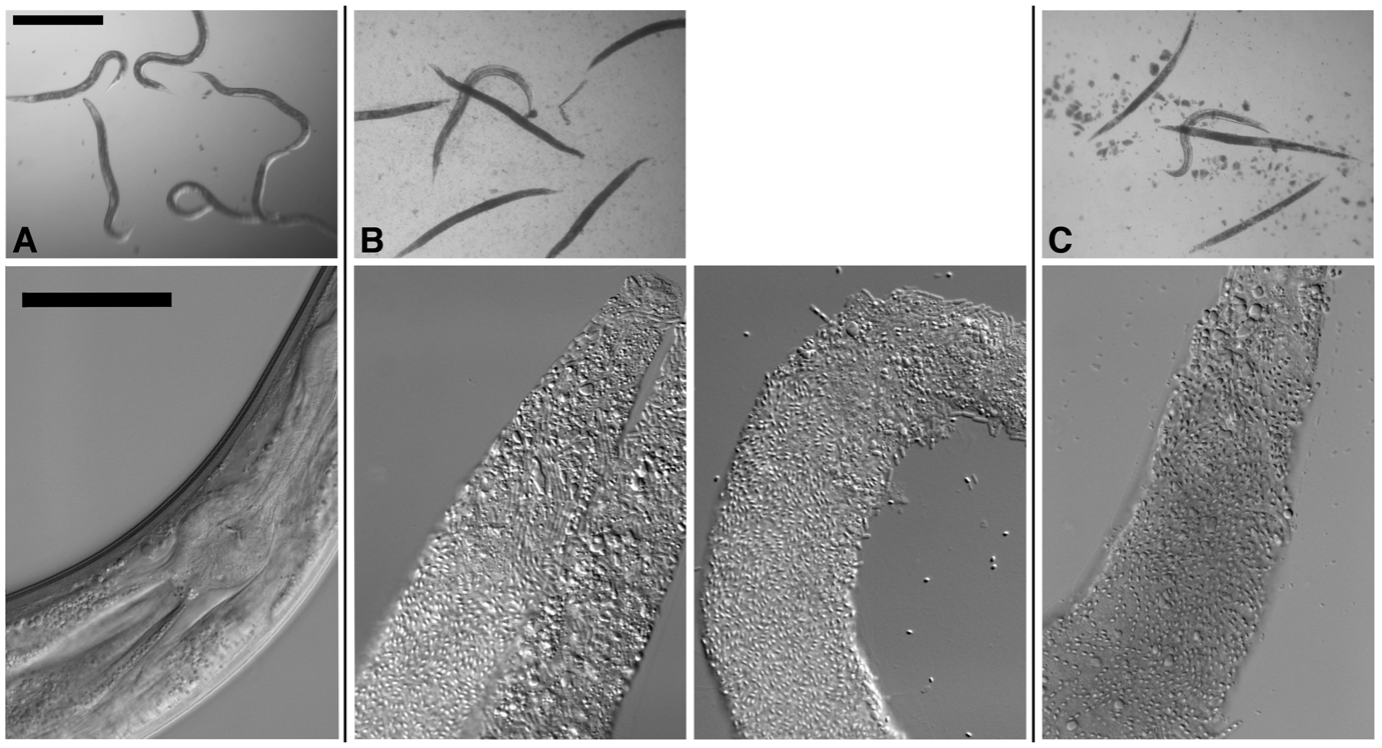
Infection of *C. elegans* by *B. thuringiensis* and *B. anthracis*. Top row: Dissecting microscope view of nematodes cultured under various conditions. Scale bar of all images in top row is 500 µm. Bottom row: Compound microscope view of nematodes cultured under various conditions. For all images in the bottom row, anterior of the worm is top right and scale bar is 50 µm. (A) *C. elegans* cultured in a well with *B. thuringiensis* without Cry5B. Top row: None of the six nematodes are infected. All are healthy. The blur associated with some of the worms in the top row is due to their movement in the well. Bottom row: The internal structures of *C. elegans* fed *B. thuringiensis* without Cry5B, including the pharynx and intestine, are all intact. (B) *C. elegans* cultured in a well with *B. thuringiensis* and Cry5B. Top row: Five of the six worms are completely infected (rigid, lack of internal structures and normal coloration); one is not. Bottom row: Infected animals show complete or near complete digestion of internal structures by the bacteria. Vegetative and sporulated bacteria can be seen in these lethally infected animals. (C) Similar images as in (B) except the bacterium cultured with the nematodes is *Bacillus anthracis*.

In our numerous studies of nematicidal Cry proteins in the absence of *B. thuringiensis* but in the presence of its standard laboratory food source, *Escherichia coli*, we have found that Cry proteins produce a dose-dependent mortality in nematodes but do not promote an *E. coli* infection. Likewise, non-Cry protein-producing *B. thuringiensis* strains, such as HD1 cry-, do not cause an infection when fed to *C. elegans* in the absence of Cry proteins (Figure 1A; Table 1; [12]). We therefore investigated what would happen if we fed *C. elegans* Cry5B, a Cry protein that forms pores in membranes [13], in the presence of *B. thuringiensis*. Purified Cry5B produces mortality in *C. elegans* over a 6 day time course at 25°C with a calculated LD_50_ of ∼8 µg/ml [14]. Under these conditions, *E. coli* is used as the food source and no infections (internal multiplication of *E. coli*) are seen; the nematodes die from Cry protein intoxication. Conversely, when we fed Cry5B to *C. elegans* at 10 µg/ml in the presence of *B. thuringiensis*, we observed accelerated killing of nematodes in 24–48 h, with the internal structures of the nematodes completely digested by the proliferating bacteria (Figure 1B, Table 1). With B. *thuringiensis*, the majority of the killing takes place in the first 24 h, although we typically let the assays run for 48 h to ensure maximum killing was reached. Nematodes killed by *B. thuringiensis* with Cry5B are filled with vegetative *B. thuringiensis* cells or *B. thuringiensis* spores (Figure 1B), a phenotype we have named “Bob” for Bag of bacteria, where the undigested cuticle of the nematode represents the bag that contains the bacteria.

**Table 1:** Infectivity of *Bacillus* sp. on *C. elegans* in the presence/absence of pore-forming Cry5B.

	*N2 wild-type animals*			
	Without Cry5B PFP		With Cry5B PFP	
*Bacillus* species	Total number of animals	Number of Bobs	Total number of animals	Number of Bobs
***Bt HD1***	107	0	133	102
***B. subtilis PY79***	119	0	128	45
***B. subtilis 6051***	115	0	122	53
***B. megaterium***	132	0	149	8

	*glp-4(bn2)* mutant animals			
	Without Cry5B PFP		With Cry5B PFP	
*Bacillus* species	Total number of animals	Number of Bobs	Total number of animals	Number of Bobs
***Bt HD1***	80	0	72	68
***B. subtilis PY79***	71	0	72	5
***B. subtilis 6051***	90	0	90	7
***B. megaterium***	70	0	76	1

	*bre-4(ye13)* animals			
	Without Cry5B PFP		With Cry5B PFP	
*Bacillus* species	Total number of animals	Number of Bobs	Total number of animals	Number of Bobs
***Bt HD1***	115	0	100	0
***B. subtilis PY79***	117	0	94	0
***B. subtilis 6051***	103	0	95	0
***B. megaterium***	106	0	101	0

Quantitation of infected animals in the presence and absence of Cry5B demonstrates that the PFP is absolutely required for the infection process—in the absence of Cry5B no *B. thuringiensis* infections are seen whereas in the presence of Cry5B a very high percentage of the nematodes succumb to accelerated lethal infections (Table 1). To confirm that the infections seen are independent of internal hatching of progeny in hermaphroditic *C. elegans*, we performed similar studies using the *C. elegans* mutant *glp-4(bn2)*, which are sterile at the restrictive temperature and do not produce progeny although they do have a similar response to Cry5B as wild-type *C. elegans* [15]. Similar robust Cry5B-mediated infections by *B. thuringiensis* are found in this genetic background as well (Table 1). In these experiments and other experiments with other *Bacillus* sp., Cry5B purified from *B. thuringiensis* was added exogenously so that all the experiments could be directly compared. We have confirmed that *B. thuringiensis* HD-1 endogenously producing Cry5B also produces a robust number of Bobs (32/33) whereas an isogenic HD-1 strain of *B. thuringiensis* lacking Cry5B production does not (0/51). Thus Cry5B PFP is an essential virulence factor required for lethal infection of *C. elegans* by *B. thuringiensis*.

Cry5B PFP intoxicates and kills *C. elegans* via binding to glycolipid receptors found in the intestine [16]. To confirm that Cry5B-stimulated *B. thuringiensis* infections require the established PFP intoxication pathways, we repeated the challenge experiments in glycolipid-receptor deficient *bre-4(ye13) C. elegans* animals [16], [17]. As predicted, nematodes lacking the receptor for Cry5B are not infected by *B. thuringiensis* even in the presence of Cry5B (Table 1).

### Dependency of the infection process upon PFP dose and bacterial inoculum size

**Figure 2:**
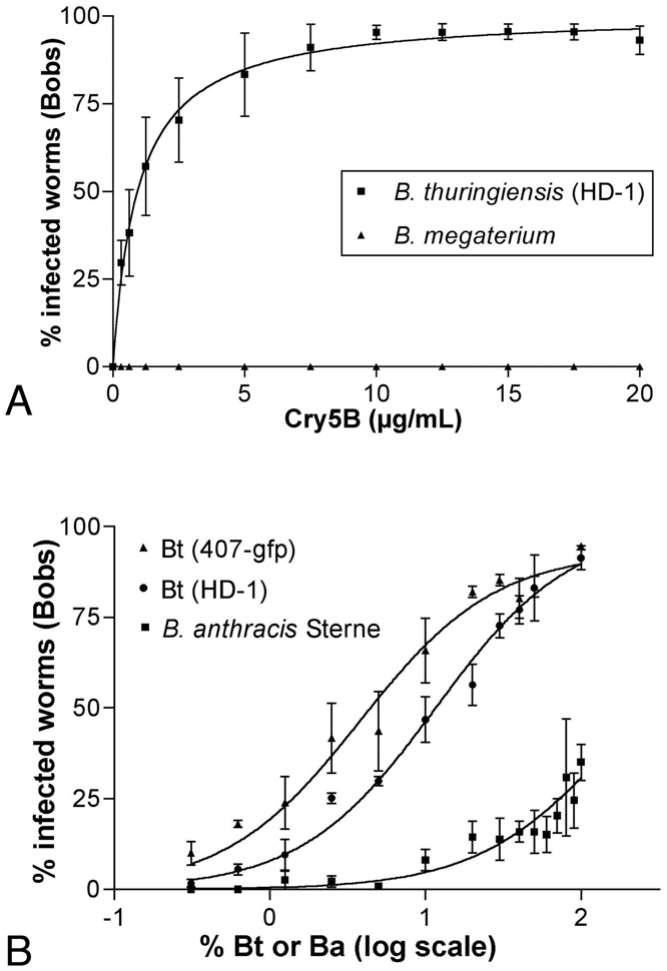
Dose-dependency of lethal infections by Bacillus and Cry5B. (A) Infections increase in a dose-dependent manner with increasing Cry5B. Doses from 0 to 20 µg/ml Cry5B were prepared and standard infection assays with *B. thuringiensis* HD-1 carried out. No worms were infected at any of the Cry5B concentrations when exposed to the relatively non-pathogenic *B. megaterium* in the negative control experiment. Error bars indicate standard error. n = 3 trials, with an average of 40 worms per condition per trial. The *glp-4(bn2)* strain was used to eliminate any concerns of internal hatching of larvae as the source of death. (B) The lethal infection rate induced by Cry5B increases with increasing doses of *B. thuringiensis* or *B. anthracis*. Varying volumes of *B. thuringiensis* or *B. anthracis* were mixed with volumes of the non-pathogenic *B. megaterium* to create different ‘doses’ of the pathogen while presenting a constant total bacterial load to the worms. Error bars indicate the SEM. n = 3 trials, with an average of 60 worms per condition per trial. The right-most data point is 100% *B. thuringiensis* or *B. anthracis*. The data for 0% *B. thuringiensis* or *B. anthracis* were collected but cannot be displayed on this log plot; there were no lethal infections at this dose.

Our experiments demonstrate that infectivity is fully dependent both upon Cry5B sensitization and the presence of a pathogenic bacterium (*B. thuringiensis*). We next set out to determine the relationship between each of these elements and their dosage. Infection assays were performed with either highly infective *B. thuringiensis* or weakly infective *B. megaterium* (as a negative control; see below) at increasing doses of Cry5B (Figure 2A). As demonstrated above, in the absence of Cry5B, no infections were seen with either bacterium (Figure 2A). As the dose of Cry5B increases, the percent of worms infected by *B. thuringiensis* HD-1 increases until maximum infectivity is reached at ∼10 µg/ml Cry5B (Figure 2A). Thus, *B. thuringiensis* lethal infection depends upon Cry5B levels in a dose-dependent manner. Because it was the smallest dose that still achieved penetrant infectivity, we utilized 10 µg/mL as our Cry5B dose in all our fixed dose experiments.

Many key epidemiological processes are inoculum-dependent, *e.g.*, the chance of infection in a given host increases with increasing pathogen inoculum (reviewed in [18]). To determine if this relationship between pathogen and host are true for *B. thuringiensis* infections in *C. elegans*, we varied the dose of inoculum by diluting *B. thuringiensis* with varying amounts of weakly infective *B. megaterium*. Two different *B. thuringiensis* strains were used for this study. We found a dose-dependent relationship in which the *C. elegans* infection rate increases when the proportion of *B. thuringiensis* in the inoculum is raised (Figure 2B). This well-behaved inoculum-dependent infectivity of *B. thuringiensis* in Cry5B-sensitized *C. elegans* is rather unique, as *C. elegans*–pathogenic bacterial studies generally focus on percent animals killed at different time points at fixed doses of bacteria versus percent animals killed at different bacterial concentrations at a fixed time.

### Temporal requirements of the infection process

**Figure 3:**
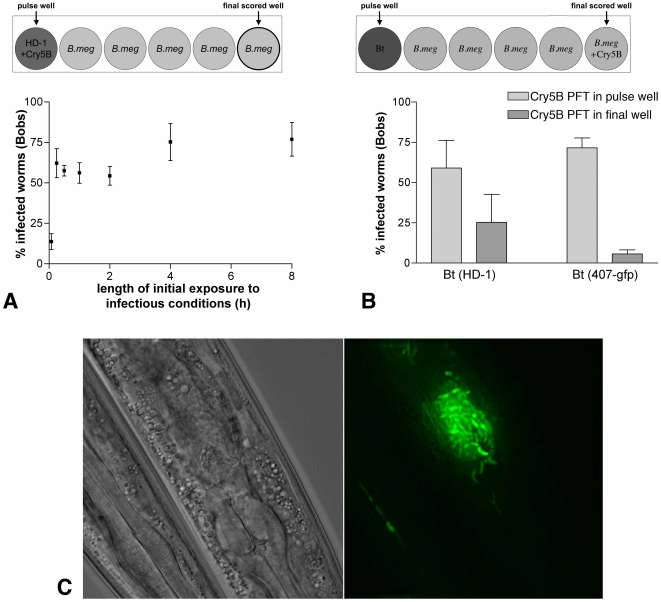
Temporal aspects of the infection process. (A) Infections are established upon a short exposure to pathogen. Upper: schematic of experiment. Worms are added to the well on the left containing *B. thuringiensis* and Cry5B. Following either 5 min, 15 min, 30 min, 1 h, 2 h, 4 h, or 8 h, the worms were then moved through a series of five wells lacking Cry5B protein and containing non-infectious *B. megaterium* instead of *B. thuringiensis*. Infection outcomes in the final well were scored 48 h later. Lower: results of the experiment depicted in upper schematic. Data shown represent a total of three independent experiments with ∼30 animals per time point per experiment. Error bars indicate SEM. Only the infection rate for a 5-minute pulse is significantly different from the other data points (ANOVA, *p*<0.05, Tukey's post test). (B) Cry5B acts early in the infection process as temporal addition of Cry5B protein after, and separate from, pathogen exposure results in a significant drop in infections. Upper: schematic of experiment in which worms are exposed to pathogen first and then to Cry5B. Lower: results in which two sets of experiments were set up simultaneously—a normal 15 minute pulse chase with both Cry5B PFP and pathogen added together (light gray bars; see (A) for set up) and pulse chase in which Cry5B was not added until the end (dark gray bars). Error bars represent SEM. ∼30 worms/condition/trial; 3–4 independent trials per condition. (C) The infection process appears to begin with colonization of the anterior intestine. The anterior intestine of a nematode 3 hr after exposure to *B. thuringiensis* (407-gfp) under pathogenic conditions. The left panel is a DIC image; the right panel deconvolved fluorescent (FITC) of the same animal. Images taken at 600×. Anterior is down.

Our data demonstrate that incubation of *B. thuringiensis* and Cry5B continually with *C. elegans* results in a lethal infection within 48 h. We next set out to study the temporal requirements for the infection process—e.g., does *B. thuringiensis* and Cry5B need to be continually fed to *C. elegans* during this entire time period or are shorter incubation periods also effective in establishing an infection? To determine when the infection process was established, a *B. thuringiensis* pulse-chase experiment was designed (Figure 3A, schematic). Young adult *C. elegans* hermaphrodites were exposed to pulses of the *B. thuringiensis* and Cry5B PFP for varying lengths of time that ranged from 5 minutes to 8 hours. Following these pulses, the nematodes were passed through a series of five washes containing *B. megaterium* (no Cry5B) to dilute out both the infectious bacteria and Cry5B. Animals were incubated in the final wash for a total of 48 hours and then scored for Bobs (Figure 3A, graph). Surprisingly, high levels of lethal infections are established in as little as 15 minutes of exposure to the pathogen plus Cry PFP. Indeed, the high levels of infections seen upon exposure to pathogenic conditions for any of the time points between 15 minutes and 8 hours are not statistically different (ANOVA, *P*>0.05, Tukey's post test).

To confirm that the infections seen in the pulse-chase experiment were not the result of residual *B. thuringiensis* and Cry5B in the final well containing *B. megaterium*, the experiment was set up as above but no worms were placed in the initial *B. thuringiensis* well. The same volume as pipetted in the above experiments (without nematodes) was then passaged from the initial well through four *B. megaterium* dilution wells to the final *B. megaterium* well as above. Nematodes and Cry5B were then added to this final well. No infections were ever detected in four repetitions of this experiment. Thus, the amount of *B. thuringiensis* transferred from the first to the final well is not sufficient on its own to cause infection and the infections seen in Figure 3 are the result of interactions between the worms and bacteria exposed to one another in the first well.

It is known that the intestinal pores formed by a short pulse of Cry5B on *C. elegans* can last for up to 1 d [19]. Thus, it is possible that the effects of Cry5B PFP on the infection process occurs late even though the pulse used in the above experiments was early and brief. To address the issue of whether Cry5B PFP is acting late or early, we first exposed the animals to the infectious bacteria, washed these away, and then added Cry5B (Figure 3B, schematic). If Cry5B were only acting late in the process, we rationalized that there should be no difference between adding Cry5B with the bacteria in the very beginning or adding Cry5B after a slight delay of ∼10 min. In fact, we found that adding Cry5B subsequent to the bacteria leads to a significant drop in infection levels. These data indicate that the PFP instigates infection of *B. thuringiensis* in *C. elegans* at the earliest stages.

To see if we could detect visual cues of the beginning of the infection process, green-fluorescent protein (GFP)- expressing *B. thuringiensis* was fed to *C. elegans* along with Cry5B PFP. The earliest and most common phenotype we could see (∼3–6 h after initiation of the experiment) was the formation of a cluster of vegetative bacteria in the interior lumen of the intestine in many of the nematodes (Figure 3C). These data suggest that, at least by this time-point, the focus point of the infection process is in the anterior intestine, from which it likely spreads.

### Cry5B PFP potentiates the infectivity of many *Bacillus* sp

Our data demonstrate that exposure to the crystal PFP Cry5B allows *B. thuringiensis* to initiate a lethal infection in *C. elegans*. We tested that the PFP might also potentiate the infectivity of other *Bacillus* sp.. First, we tested *Bacillus subtilis*. *B. subtilis* is nonpathogenic towards *C. elegans*, and *C. elegans* have increased longevity when fed on *B. subtilis* than when fed on *E. coli* OP50, the standard laboratory *C. elegans* food source [20], [21]. We quantitated the ability of *B. subtilis* (two different strains) to infect *C. elegans* in the absence and presence of Cry5B PFP. Whereas *B. subtilis* alone is unable to infect C. elegans, this “nonpathogenic” bacterium is able to infect *C. elegans* in the presence of Cry5B PFP (Table 1). As with *B. thuringiensis*, *B. subtilis* infections occur even in the sterile *glp-4(bn2) C. elegans* background (Table 1). Thus, internal hatching of progeny is not required for infection by *B. subtilis*, although some contribution may be reflected by a drop-off in infection rate in *glp-4(bn2)* animals (Table 1). As with *B. thuringiensis*, Cry5B intoxication via the glycolipid receptor is required for subsequent infection by *B. subtilis*, as *bre-4(ye13)* receptor-less mutants are not infected by *B. subtilis* in the presence of PFP (Table 1).

**Figure 4:**
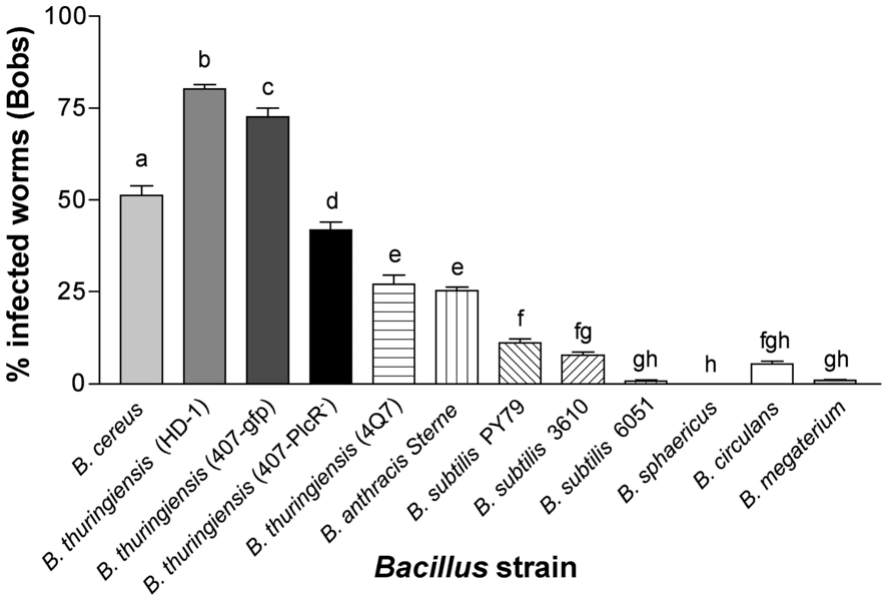
Infection of *C. elegans* by different *Bacillus* species. The percent-infected worms by different Bacillus are shown along with standard error. Means below the same letter were not significantly different at *P<0.05*; means below different letters are significantly different at *P<0.05*. Each bar represents the mean for 3–4 independent trials. The total number of animals screened from left to right are: 117, 209, 117, 122, 107, 142, 96, 96, 96, 72, 96, and 216.

To look closer at the specificity on the *Bacillus* species, we repeated these Cry5B PFP-mediated infection assays using a broader range of *Bacillus* sp. for these experiments and the *C. eleg*ans strain *glp-4(bn2)* to avoid any complication in interpretation from internal hatching of larvae (Figure 4). We found that the bacteria from the *B. cereus* group- *B. cereus*, *B. thuringiensis* 407-*gfp*, *B. thuringiensis* 4Q7 and *B. anthracis* Sterne are the most virulent, each infecting >25% of the nematodes (Figure 4). As reported in Table 1, *B. subtilis* is able to infect at an appreciable level and *B. megaterium* at a barely detectable level (Figure 4). Interestingly, *B. circulans*, which is phylogenetically quite divergent from the other Bacillus, is also capable of infection, but *B. sphaericus*, which is used as an insecticidal bacterium like *B. thuringiensis*, is not (Figure 4). *B. anthracis*-infected nematodes look similar to *B. thuringiensis*-infected nematodes under low and high-magnification microscopy (Figure 1C).

It has been previously shown that *B. thuringiensis* is capable of causing lethal infections in insects and that this infectivity is greatly dependent on the master regulator of virulence, PlcR [22], [23]. In *Galleria mellonella* larvae, co-ingestion of the *B. thuringiensis* parental strain with a sublethal concentration of Cry1C toxin caused 70% mortality whereas only 7% mortality was recorded if a Δ*plcR* mutant strain were used [23]. Interestingly, in Cry5B-sensitized *C. elegans*, loss of PlcR from *B. thuringiensis* still produces a robust, albeit reduced, percentage of infected dead animals (Figure 4; 44% of Bobs with *B. thuringiensis* 407Δ*plcR versus* 73% with the *B. thuringiensis*-407-*gfp* parental strain). Moreover, *B. anthracis* Sterne, which lacks a functional PlcR [22], is able to produce a level of infection in Cry5B-sensitized *C. elegans* comparable to one of the *B. thuringiensis* strain (4Q7) (Figure 4). PlcR is therefore dispensable for infection in *C. elegans* and much less important for infection in *C. elegans* than in *Galleria*. Taken together, Cry5B PFP is able to potentiate the infectivity of many, but not all, Bacillus towards *C. elegans*, with the tightly knit *B. cereus* group as the most potentiated.

### *B. anthracis*, which naturally lacks crystal proteins, can infect in the presence of Cry5B-*B. thuringiensis*

By definition *B. thuringiensis* is the bacterium that encodes for and produces Cry proteins whereas none of the other *Bacillus* sp., including the other *B. cereus* family members, do. We therefore wondered whether there might be any instance in which a Cry protein-minus Bacillus bacterium, such as *B. anthracis*, might be able to infect *C. elegans* with the help of *B. thuringiensis*. This question is important because it is still a mystery how *B. anthracis* maintains its life cycle in the soil so that it can be periodically transmitted to herbivore hosts [7], [8], [24]. We hypothesized that *B. anthracis* might be able to infect *C. elegans* if it was present coincidently with *B. thuringiensis*—i.e. *B. anthracis* could cause a super infection when *B. thuringiensis* was present.

**Figure 5:**
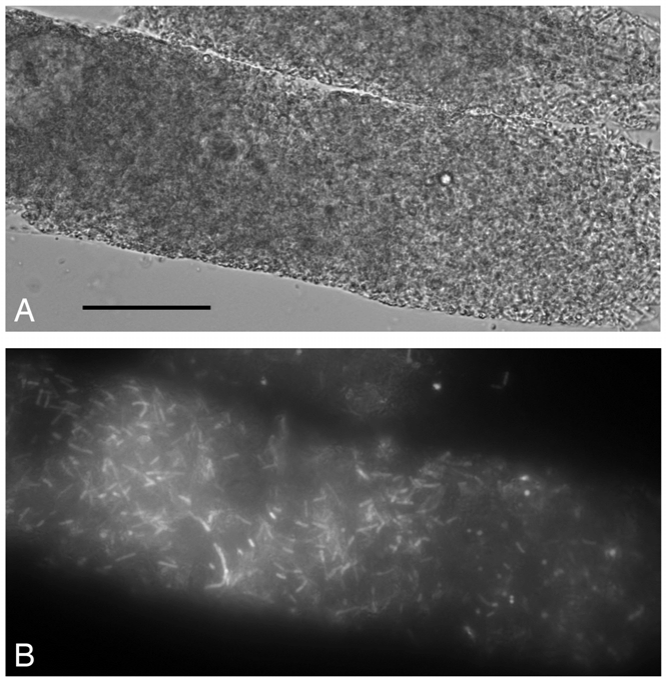
Infection of *C. elegans* by *B. anthracis-gfp* in presence of *B. thuringiensis* expressing Cry5B spores (non-*gfp*) and *B. anthracis-gfp* spores. (A) Transmitted-light view of the worm cuticle filled with vegetative and sporulated Bacillus. (B) View of the same worm using the fluorescence light: rod-shaped bacteria expressing GFP are visible, indicating that *B. anthracis* is capable of infecting *C. elegans* in presence of Cry5B expressing-*B. thuringiensis*. In this animal and all similar animals examined, the vast majority of bacteria inside the nematode cuticle were GFP positive. Scale bar is 50 µm.

We took a *B. thuringiensis* strain with a Cry5B plasmid, as might be found in the soil, and sporulated it to express Cry5B. We then mixed these *B. thuringiensis* spore-crystal lysates with sporulated *B. anthracis* expressing green-fluorescent protein (GFP). In these experiments, the bacteria were mixed at a ratio of about 16 *B. thuringiensis* spores for one *B. anthracis*-GFP spore. After 48 h of incubation at 25°C, we counted the number of Bobs and looked at them under the fluorescence microscope. We found that some of these infected worms were full of the *gfp*-anthrax (Figure 5; Table 2). Thus, even with an initially much larger load of *B. thuringiensis* being given to the animals and at a temperature (25°C) where *B. thuringiensis* might be expected to perform better, on occasion *B. anthracis* is able to overtake and drive the infection process.

**Table 2:** Infectivity of *B. anthracis* in the presence of Cry-plus B. *thuringiensis.*

Condition	Number animals screened	Number Bobs seen	No. GFP-filled Bobs
*B. anthracis-gfp*	110	0	0
*B. anthracis-gfp* plus Cry5B-*Bt*	281	72	19

## Discussion

Here for the first time we characterize in detail the infectivity of *Bacillus* family bacteria in a nematode, *C. elegans*. Beginning with the invertebrate-specific pathogen, *B. thuringiensis*, we find that the infection of *C. elegans* by *B. thuringiensis* is dependent upon the presence of a PFP crystal protein virulence factor, Cry5B. The Cry5B pore-forming protein either can be added exogenously or can be naturally expressed by *B. thuringiensis* in order to elicit infection. The *B. thuringiensis* strains used in this study cannot infect the in the absence of added Cry5B (either exogenous or endogenous). Infectivity is proportionally dependent upon inoculum size and dose of the pore-forming protein. Although the fact that *B. thuringiensis* (but not other *Bacillus* species) can infect *C. elegans* has been seen before [25], [26], the dependence of this infection on crystal protein has not been studied. We furthermore demonstrate that the PFP acts early in the process to instigate infection. The process is likely to be distinct from *B. thuringiensis* infections characterized in insects since the dependence upon the PlcR virulence regulator, which has been shown to be central for infectivity in an insect [23], is minimal in the nematode system.

Surprisingly we find that many *Bacillus* species also possess the ability to infect *C. elegans* in the presence of Cry5B. This list of *Bacillus* sp. that can infect *C. elegans* includes *B. subtilis*, a bacterium considered non-pathogenic to *C. elegans* and even more benign than the standard laboratory *E. coli* fed to it [20], [21]. Of all *Bacillus* sp. tested, *B. cereus* group bacteria show the highest level of infectivity in the presence of the Cry5B PFP. The relative role of Cry toxin to various *Bacillus* species has also been investigated in the larvae of the greater wax moth (*G. mellonella*). In this insect Cry1C toxin induced higher mortality to *B. thuringiensis* and *B. cereus* strains and to a much lower level to *B. subtilis* and *B. megaterium* and not at all with *B. anthracis* [23], [27], suggesting that *B. anthracis* develops better in nematode than in insects. We speculate that infectivity of nematodes is an ancient hallmark of the *Bacillus* genus and that either many *Bacillus* sp. have subsequently lost the ability to infect nematodes or in the wild factors other than crystal proteins (e.g., environmental stress, starvation of the nematodes, virulence factors not normally expressed in the laboratory) instigate infection.

This work establishes the Bacillus-Cry5B-*C. elegans* interaction system as a model for studying specific requirements of the nematode infection process of *Bacillus*, most notably *B. cereus*-family bacteria such as *B. anthracis*, for studying the role of PFPs in promoting bacterial pathogenesis, and for studying host responses to *B. cereus* family bacteria. The fact that both the bacteria and the host are amenable to genetics makes it possible to investigate the interactions of *B. cereus* family bacteria and an animal host at a level not previously attained.

This research also begins to address an important question in the *B. anthracis* field—what are the potential reservoirs of this bacterium in nature? *B. anthracis* causes periodic, large-scale episodes of infection in ruminants. The question has remained: where does it reside and how does it propagate between episodes? One suggestion is that earthworms are reservoirs for *B. anthracis* and *B. cereus* multiplication and persistence [28]. Our data suggest that nematodes are potential targets too, when present with *B. thuringiensis*, *B. anthracis* is able to replicate within a nematode. Therefore, nematodes might provide a key, and heretofore elusive, natural reservoir for *B. cereus* and *B. anthracis* along with earthworms and could play a specific epidemiological role in anthrax outbreaks in the wild. Furthermore, nematodes may provide a key ecological niche linking the three bacteria in the *B. cereus*-group and facilitating genetic exchange, such as the many that have already been observed [29], [30] and thus helping shape the common evolution of these bacteria.

## Materials and Methods

### Bacterial strains

Bacteria were cultured in Brain Heart Infusion (BHI) media (BD, Sparks, MD, USA) at 30°C (37°C for *B. anthracis* Sterne). For sporulation, *B. anthracis* was cultured in Difco Sporulation Medium [31]. The *Bacillus* strains used in this work were: *B. cereus* (ATCC 14579), *B. thuringiensis* (HD-1), *B. thuringiensis* (407-gfp), *B. thuringiensis* (407-ΔPlcR-gfp), *B. thuringiensis* (4Q7), *B. anthracis* Sterne, *B. anthracis* 7702 harboring pUTE610 (a gift from Theresa Koehler, University of Texas), *B. subtilis* PY79 (a gift from Richard Losick, Harvard University), *B. subtilis* 3610, *B. subtilis* 6051, *B. sphaericus* (ATCC 4525), *B. circulans* (ATCC 4513), and *B. megaterium* (ATCC 14581). *B. thuringiensis* 407-gfp and *B. thuringiensis* (407-ΔPlcR-gfp) contain a constitutively expressed Mut1-green fluorescent protein (GFP) carried on a multicopy plasmid (pHT315 *papha3::gfp*) with an erythromycin selection factor [32].

### Nematode strains

*C. elegans* were maintained using standard techniques [33]. Unless otherwise mentioned, *glp-4(bn2)* worms were used in all assays to prevent matricidal death due to premature hatching of embryos. At 25°C, this strain lacks germline and does not produce progeny. This strain is often used in *C. elegans* assays involving bacterial pathogens [15], [34], [35], [36], [37], [38]. We found that many fertile *C. elegans* hermaphrodites can die from the internal hatching of larvae when exposed to *Bacillus* spp. under conditions of the infection model. Invariably, these dead worms would subsequently become infected. Under these circumstances, it was not easy to differentiate between worms that had become infected before dying versus worms that had died from internal hatching of larvae before becoming infected. Thus, we use *glp-4(bn2)* in many of our assays to avoid this ambiguity. We do not believe the use of *glp-4(bn2)* significantly alters our findings. Similarly robust infections are achieved using wild-type N2 *C. elegans* as with *glp-4(bn2)*, infected animals look the same under high power magnification regardless of which strain is used, and a completely different sterile strain, *fer-1(ba576)*, shows similar levels of infection to *glp-4(bn2)* (data not shown). *glp-4(bn2)* worms were maintained at 15°C and shifted at the appropriate times to 25°C when sterility was desired.

### Expression and Purification of Cry5B protein

Purified Cry5B protein was prepared using a modified sucrose gradient [39]. Purified Cry5B protein was precipitated, resuspended in double-distilled H_2_O, frozen into aliquots using liquid nitrogen, and stored at −80°C until needed. On the day of use, aliquots were thawed at room temperature and centrifuged at 16,000 *g* to pellet the protein and allow aspiration of the supernatant. Pellets were then solubilized in 20 mM citrate buffer (pH 3.0) to a final concentration of 0.4 mg/ml.

### *Bacillus* infection assays

Unless otherwise specified, infection assays were carried out in 48-well assay plates. The final volume in each assay well was 200 µl. Standard S-medium was used to maintain *C. elegans* in liquid cultures [33].

#### Preparation of bacteria

Bacteria were grown overnight in 5 ml liquid BHI in a standard large test tube (25×150 mm) at 250 rpm, 30°C (37°C for *B. anthracis*). The overnight culture was then centrifuged in 1.5-ml tubes in a microcentrifuge at 16,000 *g* for 4 min to pellet cells and the supernatant was aspirated. Bacterial cells were resuspended in S-medium for a final OD_600_ of 0.3+/−0.02. 190 µl of bacteria in S-media was added to the assay well. This methodology allows for relatively easy normalization amongst the different bacterial strains.

For assays with mixing of *B. anthracis* and Cry5B-expressing *B. thuringiensis*, *B. anthracis-gfp*, spores were used since *B. thuringiensis* produces Cry5B during sporulation. Bacteria were grown in 5 ml liquid Difco Sporulation Medium for 48 hours at 37°C [31]. Cultures were checked using a compound microscope for sproulation and were found to be >99% spores. 0.1 µl of Cry5B spore crystal lysates, containing about 4.0×10^6^ spores were added with 10 µl of *B. anthracis-gfp* spores (containing about 2.5 10^5^ spores) to 190 µl of S-media in a 48-well plate. *glp-4(bn2*) animals were prepared as described below.

#### Addition of Cry5B protein

5 µl of 0.4 mg/ml Cry5B in 20 mM citrate buffer (pH 3.0) was added to appropriate wells for a final well concentration of 10 µg/ml Cry5B. Citrate buffer (pH 3.0) alone was used as a negative control.

#### Preparation of nematodes

Synchronized populations of *glp-4(bn2)* nematodes were prepared using standard techniques [39]. Two days prior to setting up the infection assay, a synchronous population of L1s was seeded onto standard 3.5-cm NG plates containing OP50. The worms were incubated at 25°C for 52 h to sterilize the hermaphrodites and allow them to reach the young adult stage. The synchronized nematodes were then washed off the plates with M9 and rinsed three times; twice with M9 and the final time with S-medium. Worms were centrifuged at 500 *g* for 40 sec to pellet worms for removal of wash supernatants. Worms were then resuspended in S-medium for the desired concentration of 8–12 worms per 5 µl, and 5 µl of this suspension was then added to wells. The assay plates were incubated at 25°C for 48 h, after which they were scored for dead infected worms with a dissecting microscope. Although the majority of nematodes that would end up infected were infected and dead in one day, the assays were allowed to go for two days to be sure the infections had gone to completion. Worms were only counted as infected worms if they were filled with bacteria, which can be detected by dark, rigid, and immobile worms. We have confirmed numerous times by high-resolution microscopy that such worms represent terminally infected worms.

### Cry5B dose-response assay

Preparation of bacteria and worms was identical to the described procedure of the *C. elegans* infection assay. Different doses of the Cry5B PFP were prepared by serial dilution so that the same volume of protein would be added to each well. Each condition was tested in multiple wells (from 4–6 wells).

### *Bacillus* dose-response assay

Bacteria were prepared in S-medium according to the *C. elegans* infection assay protocol. Different doses of pathogenic *Bacillus* were prepared by mixing varying amounts of pathogenic *Bacillus* (i.e. *B. thuringiensis* HD-1 or *B. thuringiensis* 407-gfp) with the non-pathogenic *B. megaterium*, all at the same OD_600_. The inoculum amount of pathogenic *Bacillus* was expressed as the percentage of the mixed bacteria by volume. These doses were pipetted into appropriate wells and the rest of the assay was set up per the *C. elegans* infection assay protocol. Each condition was tested in quadruplicate wells.

### *Bacillus* pulse-chase assay

This pulse-chase assay was designed to test the minimum length of time needed for the bacteria to establish an infection. To allow for ease of manipulation, 24-well plates were used. Each row was dedicated to one condition. The total volume in each assay well was 500 µl. The *Bacillus* strains, purified Cry5B, and nematodes were prepared in an identical manner to the *Bacillus* infection assay. The first well of each row was designated the ‘Pulse’ well and contained 475 µl pathogenic *Bacillus* and 12.5 µl purified Cry5B protein (final concentration 10 µg/ml). The remaining 5 wells of each row were designated the ‘Chase’ wells, each containing 450 µl non-pathogenic *B. megaterium*. At t_0_ 12.5 µl of *glp-4* young adults in S-media (approximately 40 worms) were added to the ‘Pulse’ well and the plate was incubated at 25°C for 5 min, 15 min, 30 min, 1 h, 2 h, 4 h, or 8 h. At the end of the exposure time, the worms were serially transferred through the ‘Chase’ wells in a volume of 50 µl (spending less than 1 min in each intermediate well), such that the final chase well had a total volume of 500 µl. During the chase transfers, the plates were kept tilted at a 60° angle on the bench top to allow worms to more rapidly settle to the bottom and collect them for pipetting to the next well. Plates were then returned to 25°C. At t_48_ the final chase wells were scored for dead infected worms. Worms that were not transferred in the 50 µl serial washes and thus did not make it into the final ‘Chase’ well were excluded from analysis.

### Microscopy

Images of worms in wells were taken with a Nikon Coolpix digital camera on an Olympus dissecting microscope. Images of the infection process were captured using differential interference contrast (DIC) and fluorescence optics on a Zeiss AxioImager A1 microscope using an AxioCam HRm camera and a 63×1.4 NA PlanApochromat lens. Images of colony formation were taken on an Olympus IX70 DeltaVision microscope (Applied Precision, Issaquah, WA) with a Nikon 60×, 1.4 NA PlanApo oil objective lens. Nematodes were mounted on glass slides with 2% agarose pads with 15 mM sodium azide as an anesthetic prior to imaging. Images were recorded and deconvolved using the SoftWorx program (Applied Precision).

### Statistics

Statistical analyses were performed with GraphPad Prism (GraphPad Software, San Diego). For multiple-group comparisons, ANOVA was performed followed by Tukey's posttest. Values of *p*<0.05 were considered statistically significant.
